# Correlation between multi-drug resistant organisms and antimicrobial use among in-hospital patients at a tertiary hospital in the Philippines from July 2010 to June 2014

**DOI:** 10.1186/2047-2994-4-S1-P171

**Published:** 2015-06-16

**Authors:** RK So, MM Mendoza

**Affiliations:** 1 Internal Medicine, Cardinal Santos Medical Center, San Juan City, Philippines

## Introduction

Infections caused by multidrug resistant organisms (MDRO) are associated with higher morbidity, mortality and healthcare costs. WHO recommends hospitals to monitor antimicrobial use to reduce MDRO prevalence.

## Objectives

This 4-year study described the annual MDRO prevalence and annual antimicrobial consumption. It also investigated their relationship.

## Methods

Annual antibiogram of Methicillin Resistant *S.aureus*(MRSA), ESBL *E.coli*, ESBL *K.pneumoniae*, and MDR *P.aeruginosa* were evaluated. Data on annual consumption (Defined Daily Dose) of selected antimicrobials were analyzed. Linear regression was used to analyze trend in antimicrobial consumption and MDRO prevalence. Pearson’s correlation coefficient was used to determine their relationship. A p-value<0.05 and r^2^>0.5 were considered statistically significant.

## Results

There was a significant increase in annual patient days while annual antibiotic usage decreased. The most common antibiotic class used was cephalosporin, followed by beta-lactam/beta-lactamase inhibitors then fluoroquinolones. Individually, piperacillin-tazobactam, ceftriaxone and ertapenem use significantly increased. The prevalence of ESBL *E.coli* significantly increased, ESBL *K.pneumoniae* and MRSA remained stable and MDR *P.aeruginosa* significantly decreased. The increased consumption of cefazolin, cefepime, meropenem and cotrimoxazole were positively correlated with increased ESBL *E.coli* prevalence. The higher use of antimicrobials without anti-Pseudomonal activity ceftriaxone and ertapenem versus piperacillin-tazobactam were positively correlated with decreased MDR *P.aeruginosa* prevalence. MRSA prevalence positively correlated and mirrored linezolid usage as it is a 2^nd^ line agent for it. The absence of increase in ESBL *K.pneumoniae* prevalence may be due to decreased fluoroquinolone use.

## Conclusion

This study at our institution found that antimicrobial use did not increase despite increase in annual patient days. The prevalence of ESBL *E.coli* increased, ESBL *K.pneumoniae* and MRSA remained stable; MDR *P.aeruginosa* decreased. A positive correlation between antimicrobial use and MDRO prevalence was established. Establishing an antibiotic restriction program is recommended to address the significant prevalence of MDRO.

## Disclosure of interest

None declared.

